# Intraseasonal variation of the summer rainfall over the Southeastern United States

**DOI:** 10.1007/s00382-018-4345-6

**Published:** 2018-07-06

**Authors:** Wei Wei, Wenhong Li, Yi Deng, Song Yang

**Affiliations:** 1 School of Atmospheric Sciences, Sun Yat-sen University, Guangzhou, China; 2 Earth and Ocean Sciences, Nicholas School of the Environment, Duke University, Durham, NC 27708-0328 USA; 3 Guangdong Province Key Laboratory for Climate Change and Natural Disaster Studies, Sun Yat-sen University, Guangzhou, China; 4 School of Earth and Atmospheric Sciences, Georgia Institute of Technology, Atlanta, GA USA

**Keywords:** Intraseasonal variation, Southeastern United States summer rainfall, North Atlantic subtropical high, Low-level jet

## Abstract

This study characterizes the intraseasonal variability (ISV) in the Southeastern United States (SE US) rainfall in boreal summer and delineates the associated dynamical processes featuring three-way interactions among the SE US rainfall, the central US low-level jet (LLJ), and the North Atlantic subtropical high (NASH). The analysis reveals that the ISV of the SE summer rainfall peaks at the 10‒20-day timescales. The physical mechanisms for the three-way interactions on the 10‒20-day timescales are proposed. When the NASH attains a minimum strength, the reduced size of the NASH is accompanied with an eastward retreat of the western ridge of the NASH, leading to a decrease in the zonal pressure gradient and consequently a weakening of the LLJ 1 day after. The weakened LLJ and the eastward-shifted NASH western ridge induces anomalous cyclonic circulation over the SE US, moves preferred regions of moisture convergence from central US to the SE US, and 3 days later the SE US rainfall attains its maximum strength. The excessive latent heating associated with the enhanced SE US rainfall excites an anomalous anticyclone northeast of the rainfall region, resulting in an increase in the NASH intensity that peaks 2 days after the maximum SE US rainfall. The NASH subsequently expands with its western ridge moving westward, zonal pressure gradient restored, and LLJ strength recovered. An anomalous anticyclone then emerges over the SE US and suppresses rainfall, marking the shift from an intraseasonal wet phase to dry phase in this region. A more rigorous proof of these causalities demand carefully designed numerical experiments and further statistical analysis in future. Our results suggest that improved prediction of SE US summer rainfall across intraseasonal scales depends critically on the model representation of the three-way coupling among the NASH, the central US LLJ, and the SE US rainfall.

## Introduction

**Fig. 1:**
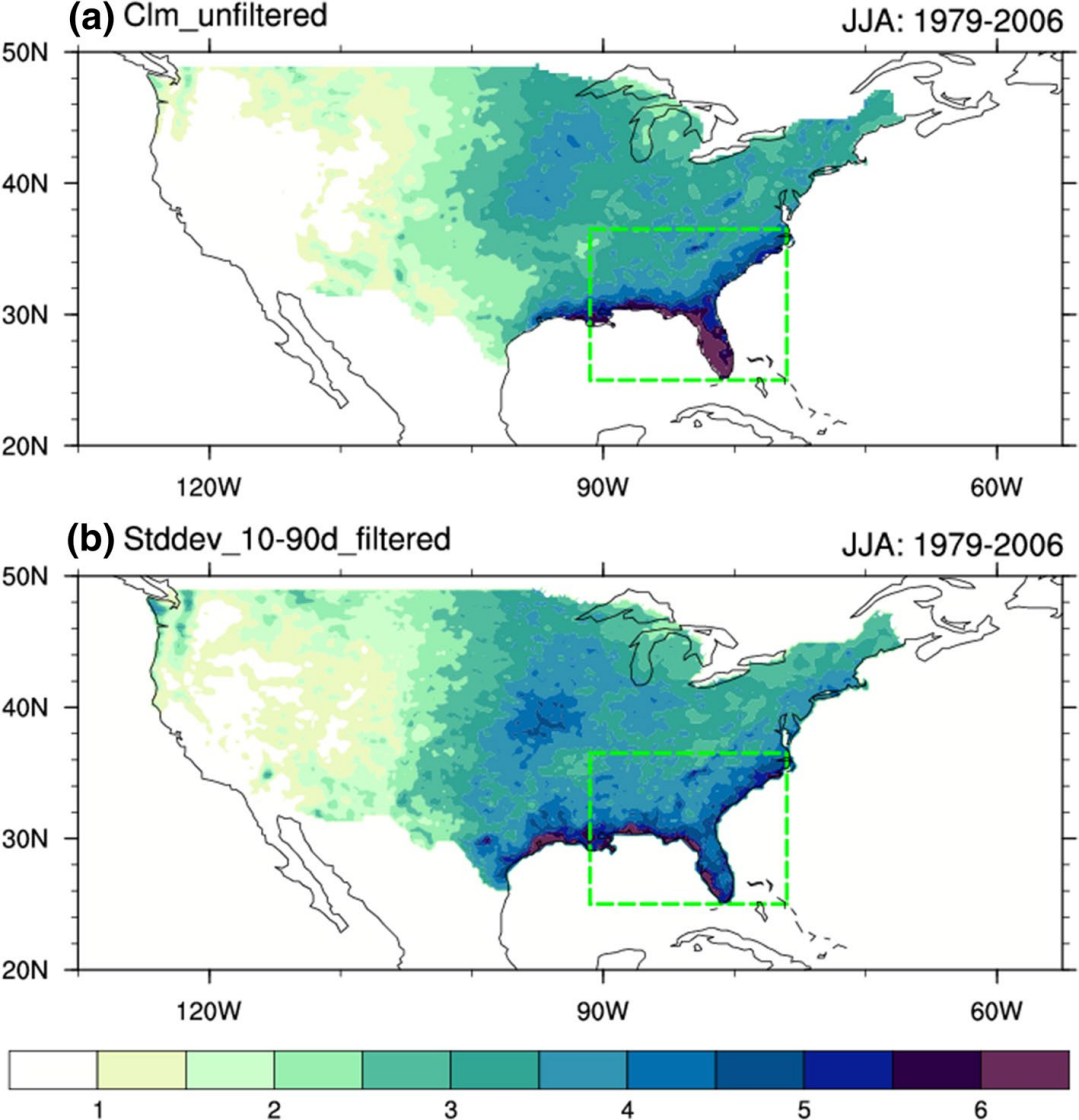
**a** Climatological summer rainfall over the contiguous United Stated (shaded; mm/day) and **b** standard deviation of 10‒90-day filtered rainfall (shaded; mm/day) in JJA from 1979 to 2006. Green dashed boxes indicate the SE US region

During boreal summer, abundant summer rainfall with high intraseasonal variance is observed over the Southeastern United States (SE US) (Fig. 1). The anomalous summer rainfall, especially the extreme precipitation events, exerts a great impact on regional hydrology, agriculture, and economics in the SE US. Thus, an accurate prediction of summer rainfall is very important for the SE US. However, the rainfall over the southeast, which is closely related to the internal atmospheric variability (Seager et al. 2009) and is affected by different weather systems and synoptic events, owns a low predictability in summer (Infanti and Kirtman 2014). It is of great significance to ascertain a clear qualitative linkage among different climate systems. Understanding the characteristics and evolution of the intraseasonal variation (ISV) of SE US summer rainfall is helpful for improving extended range prediction.

Intraseasonal variation, mainly including the 30‒60-day Madden-Julian Oscillation (Madden and Julian 1971, 1972; Zhang 2005) and the 10‒20-day quasi-biweekly oscillation, has been well investigated in the tropics (Wheeler and Hendon 2004; Jiang and Li 2005; Ling et al. 2012; Zhao et al. 2013), the monsoon regions (Krishnamurti and Bhalme 1976; Krishnamurti and Ardanuy 1980; Lau et al. 1988; Chen and Chen 1993; Qi et al. 2008; Kikuchi and Wang 2009; Wen et al. 2010), and the mid-high latitudes (Ding and Wang 2007; Wang et al. 2013; Yang and Li 2016a). In the Western Hemisphere, much effort has been devoted to understanding the ISV in the eastern North Pacific (Jiang and Waliser 2009), West Africa (Janicot and Sultan 2001; Sultan et al. 2003; Maloney and Shaman 2008), the tropical North America (Wen et al. 2011), and the North American monsoon region (Mo 2000). However, less evidence has been provided for the ISV feature of the SE US summer rainfall.

Previous analysis has investigated the ISV of summer rainfall over North America, but mainly focused on the rainfall over the Arizona and New Mexico (AZNM) monsoon region, the Great Plains (Mo 2000), and the central US (Helfand and Schubert 1995; Schubert et al. 1998; Walters et al. 2008; Weaver et al. 2009). Mo (2000) found that an ISV anomaly of rainfall with a period of 22‒25 days propagated eastward from the North Pacific through AZNM, the Great Plains, to the eastern US. The central US low-level jet (LLJ), the super geostrophic wind with the speed maximum below 850 hPa in a north–south orientation over the Great Plains (Bonner 1968; Paegle 1984; Stensrud 1996; Weaver and Nigam 2008, 2011), has been found to exhibit apparent intraseasonal scale variability; and the ISV of the central US LLJ affects the rainfall over AZNM and the Great Plains via modulating moisture transport (Schubert et al. 1998). Analysis of the low-level circulation and moisture flux revealed that the moisture transport was tied to slowly eastward-moving systems on synoptic (4–8 days) and longer (8–16 days) time scales in the contiguous United States (CONUS, Schubert et al. 1998). These results imply that the ISV of the SE US rainfall is vital and the LLJ over the central US (Higgins et al. 1997), located to the west of the SE US, likely plays a role in the ISV of SE US rainfall (Schubert et al. 1998).

**Fig. 2:**
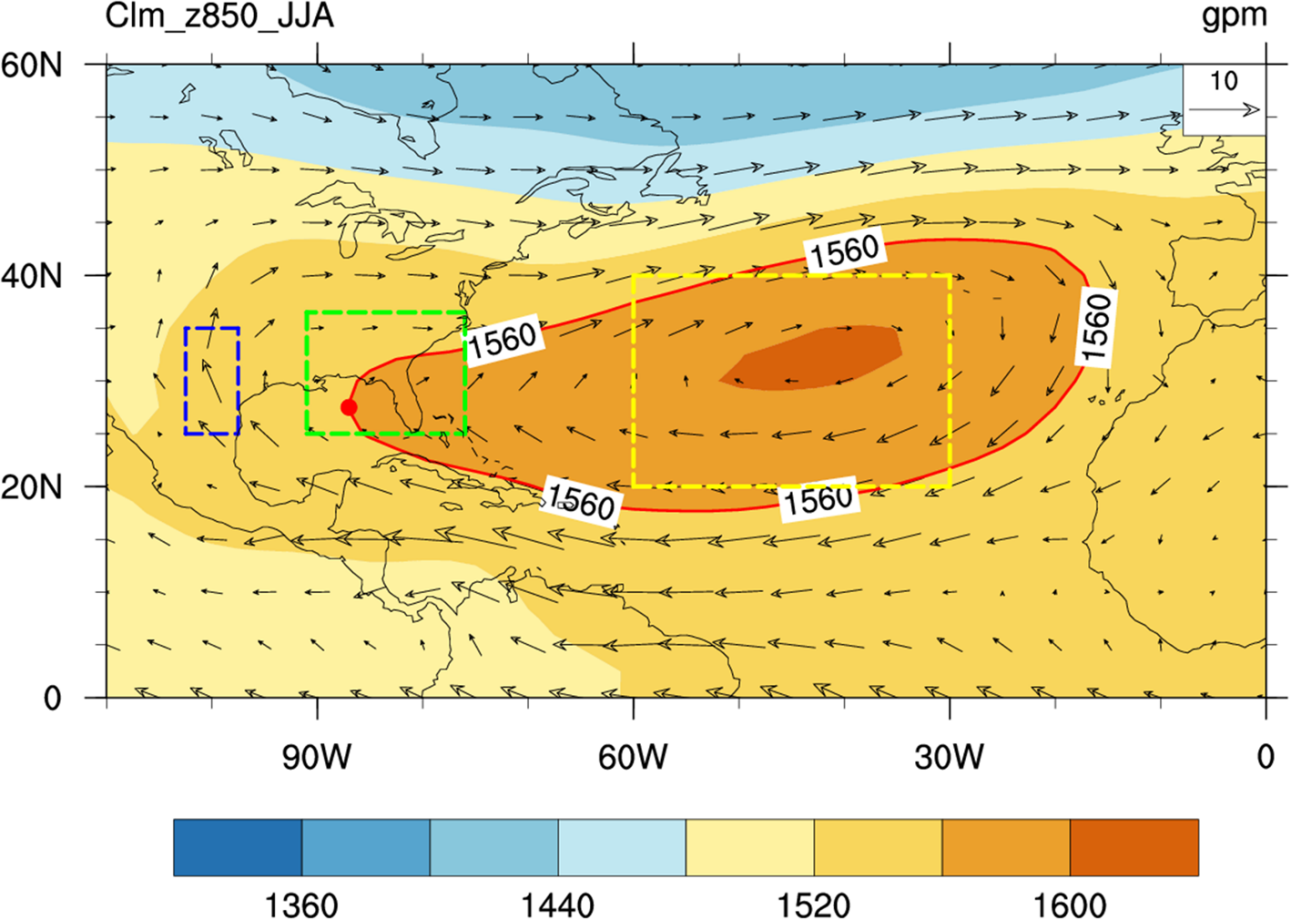
Summertime climatology of geopotential height (shaded; gpm) and horizontal winds (vectors; m s^−1^) at 850 hPa. Blue, green and yellow dashed boxes indicate the regions for the LLJ, the SE US rainfall, and the NASH intensity indices. NASH represented by 1560 isoline is highlighted by red contour. The western ridge of the NASH is shown by red dot

Rainfall over the SE US is known to be governed by the North Atlantic subtropical high (NASH), especially by the western ridge (Fig. 2) of the NASH, during boreal summer (Davis et al. 1997; Li et al. 2013). On interannual time scales, the SE US rainfall is suppressed when the western ridge of NASH is located northwest relative to its climatological mean position due to the dominate downdraft over the Southeast. When the western ridge is located southwest to its climatological mean position, moisture transport is enhanced into the region because of the abnormally strong southwesterly winds along the western flank of NASH (Li et al. 2012). Thus, the NASH plays a key role in affecting the moisture transportation and the vertical motion over the SE US (Li et al. 2011, 2012, 2013). However, on the intraseasonal scales, it is still unclear what the relationship between the evolution of the SE US rainfall and that of the NASH is.

This study attempts to seek the answers for the following questions: What are the characteristics of the summer rainfall over the SE US on intraseasonal time scales? What are the relationships among the ISV of the SE US rainfall, the central US LLJ, and the NASH? What is the physical mechanism for the interaction among SE US rainfall, the LLJ, and the NASH on the intraseasonal time scales? In the following section, data sets and analysis methods are described. Section 3 discusses the ISV feature of the SE US rainfall. The interaction processes among the SE US rainfall, the LLJ over central US, and the NASH are also investigated in this section. Conclusions are given in Sect. 4.

## Data and methods

### Data

The daily reanalysis data used in this study are extracted from the National Centers for Environmental Prediction (NCEP)/Department of Energy (DOE) Reanalysis 2 (NCEP-2), on a 2.5° × 2.5° horizontal resolution, from 1979 to 2006 (Kanamitsu et al. 2002). The CPC Unified Gauge-Based Analysis of Daily Precipitation over the contiguous United States (CONUS) is provided by the NOAA/OAR/ESRL PSD, Boulder, Colorado, USA, from its Web site at http://www.esrl.noaa.gov/psd/. The horizontal resolution is 0.25° × 0.25° US grid (20.125°‒49.875°N, 230.125°‒304.875°E) and the temporal coverage is from 1979 to 2006 (Chen et al. 2008).

### Methods

To investigate the ISV of SE US rainfall and the associated atmospheric circulation anomalies, the Butterworth bandpass filter is applied on all data from 1 January 1979 to 31 December 2006 (Butterworth 1930). Before applying the bandpass filter, the climatological mean of each day has been subtracted in order to remove the impact of the annual cycle. Finally, we select the 92 days of each year from 1 June to 31 August to investigate ISV in summer season.

Four indices are defined to effectively analyze the temporal evolutions of the Southeast summer rainfall and the interactions among the rainfall, the LLJ, and the NASH intensity and western ridge location. Following previous studies, SE US rainfall refers to the rainfall in the region 25°‒36.5°N, 91°‒76°W (Fig. 1; Wang et al. 2010; Li et al. 2011, 2012, 2013). A series of the standardized ISV rainfall index are thus obtained by the following these steps: (1) to subtract the climatological mean of each day from the daily rainfall; (2) to apply the Butterworth bandpass filter on the data from 1 January 1979 to 31 December 2006; (3) to select the 92 days of each year from 1 June to 31 August; (4) to calculate the domain-average rainfall over the SE US (25°‒36.5°N, 91°‒76°W); and (5) to standardize the rainfall series. The LLJ index is defined as the meridional wind at 850 hPa over the region 25°‒35°N, 102.5°‒97.5°W (Fig. 2; Weaver and Nigam 2008). The NASH intensity is defined as the geopotential height at 850 hPa over the NASH center region at 20°‒40°N, 60°‒30°W (Fig. 2). Finally, the western ridge of NASH is defined as the westernmost position in longitude of the isopleth 1560 gpm (Fig. 2; Li et al. 2011, 2012, 2013). Climatologically, the average NASH western ridge is located at about 87ºW in summer, a positive (negative) value of the standardized index of the western ridge longitude indicates a westward extended ridge with the west longitude greater (less) than 87ºW. The ridge-line of the subtropical high is where the winds with an easterly component reverse to the winds with a westerly component, or mathematically it fulfills that u = 0 and $\frac{\partial u}{\partial y}>0$, where u is the zonal wind component (Liu and Wu 2004).

Composite analysis is applied to summer rainfall and different atmospheric variables based on the standardized filtered SE US rainfall index to analyze the evolutions of the Southeast US summer rainfall and associated circulation anomalies on intraseasonal time scales. We define day 0 as the peaks of the standardized filtered rainfall index which are higher than 1. Day –n and day n refer to *n* days before and after the peak rainfall days (day 0), respectively. In the total 2576 days during the 28 summers from 1979 to 2006, 107 peaks are chosen as the day 0 of the extreme ISV rainfall cases in this study.

## Results

### Intraseasonal variation of the SE US rainfall

**Fig. 3:**
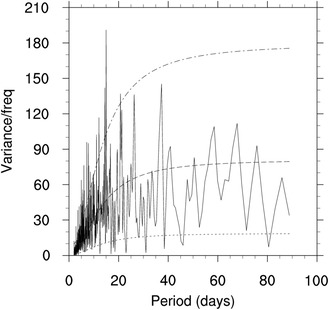
Power spectrum analysis on the unfiltered SE US rainfall. Dash-dot, dash, and dot indicate 0.95 confidence bound for Markov, Markov red noise spectrum, and 0.05 confidence bound for Markov, respectively

Climatologically, summer rainfall over the contiguous United States increases gradually from west to east with abundant rainfall over the SE US (domain-averaged rainfall is about 4.3 mm/day, Fig. 1a). On intraseasonal time scales, the standard deviation of 10‒90-day filtered summer rainfall shows the maximum values in the SE US, especially along the southeast coast of the US (Fig. 1b). Figure 1 indicates that both seasonal rainfall and its ISV are notable over the SE US. A power spectrum analysis is carried out on the SE US rainfall time series for the 28 summers from 1979 to 2006 (Fig. 3). The result reveals that besides the 2‒9-day *synoptic* variation, the 10‒20-day oscillation is significant at the 0.05 significance level, which is a dominant mode on *intraseasonal* time scales. In order to understand the characteristics and evolution of the intraseasonal variation of summer rainfall and to improve the extended range prediction over the SE US, we focus on the 10‒20-day intraseasonal time scales in this study.

**Fig. 4:**
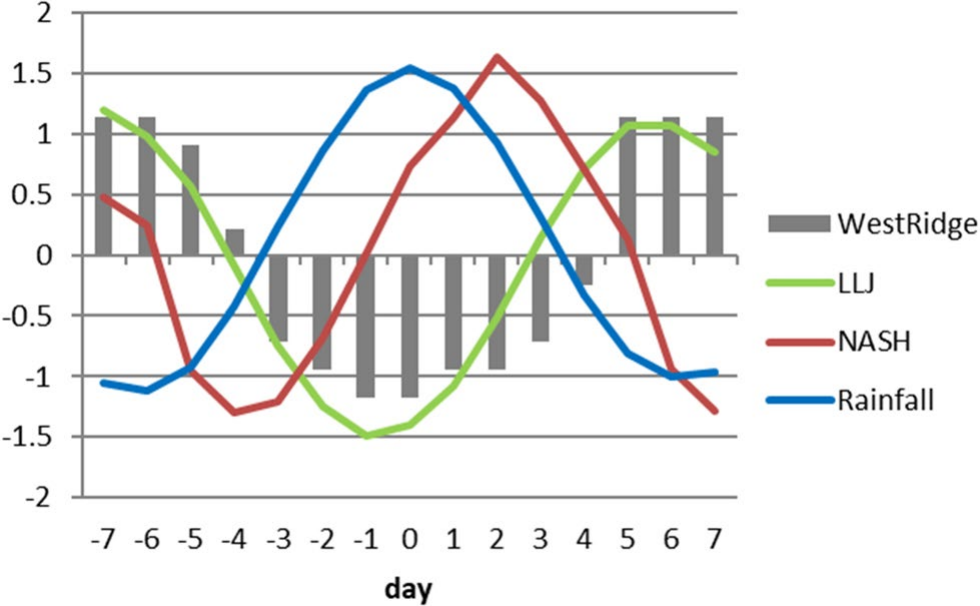
Evolutions of standardized composite 10‒20-day filtered SE US rainfall (blue line), NASH intensity (red line), LLJ (green line), and the longitude of NASH western ridge (grey bars) from day − 7 to day 7 based on the SE US rainfall index

Figure 4 shows the evolution of standardized rainfall index over the SE US on intraseasonal time scales. From day − 3 to day 3, rainfall anomalies averaged over the SE US (blue line) are positive, which can be defined as the wet phase (Fig. 4). From day − 7 to − 4 and from day 4 to day 7, rainfall anomalies are negative and are thus considered as dry phases. The maximum rainfall anomaly appears on day 0, and the minimum rainfall anomalies occur on both day − 6 and day 6 indicating a 13-day period.

**Fig. 5:**
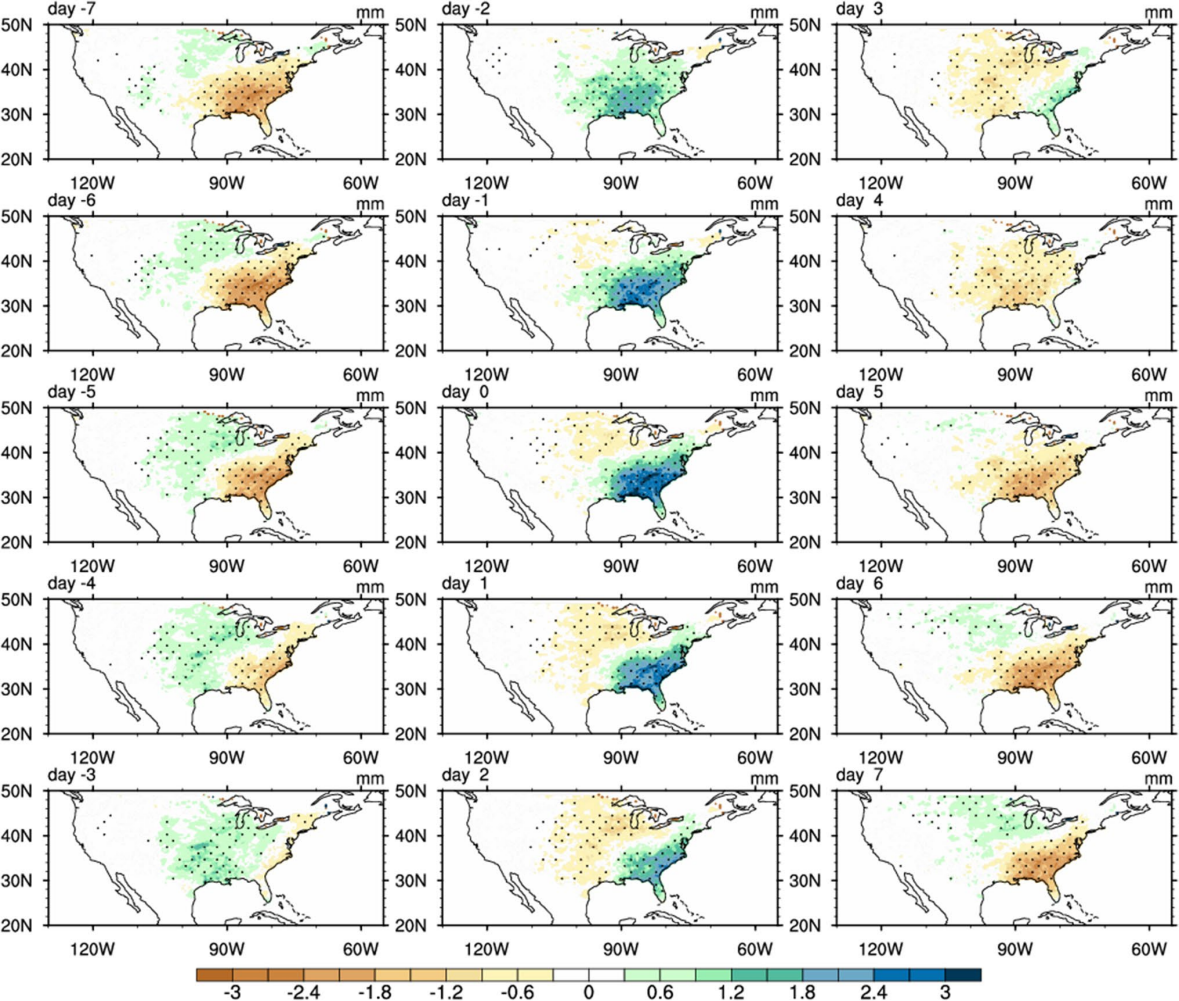
Composite patterns of 10‒20-day filtered summer rainfall anomalies (shaded; mm/day) from day − 7 to day 7 based on the SE US rainfall index. Rainfall anomalies exceeding 0.05 significance level are highlighted by dots

Figure 5 shows the composite anomalies of 10‒20-day filtered summer rainfall over the contiguous US. Spatially, negative rainfall anomalies are dominant over the SE US and they decrease gradually during the dry phase from day − 7 to day − 4 (Fig. 5). Positive rainfall anomalies appear first in the central US, and the magnitude of rainfall anomalies increases and the area of positive rainfall anomalies expand southeastward from day − 7 to day − 3. On day − 2, positive rainfall anomalies are dominant over the SE US, reaching the maximum value on day 0. Meanwhile, negative rainfall anomalies appear over the central US. From day 0 to day 3, the positive rainfall anomalies over the SE US decrease and move southeastward gradually, while the negative rainfall anomalies in the central US increase and extend eastward. From day 4 to day 7, negative rainfall anomalies are dominant in the SE US, indicating that the SE US becomes dry again. Figure 5 suggests an opposite evolution of summer rainfall over the SE US and the central US.

In summary, the quasi-biweekly oscillation of summer rainfall is a dominant mode of summer rainfall fluctuation over the SE US on intraseasonal time scales. The ISV of rainfall anomalies with a 13-day period originates from the central US and propagates southeastward to the Southeast US.

### Relationship among the NASH, central US LLJ, and SE US rainfall

Previous studies have demonstrated that the LLJ over the central US plays an important role in the summer rainfall over the central US and AZNM monsoon region on intraseasonal time scales (Mo 2000; Weaver and Nigam 2008, 2011). The coherent large-scale circulation pattern also has implications for southeastern US (Weaver et al. 2009). Besides, the summer rainfall over the SE US is found to be closely related with the variation of NASH, especially with the variability in its western ridge on interannual time scales (Li et al. 2011, 2012, 2013). Here we will discuss the temporal evolutions of the central US LLJ, the NASH, and their relationships with the ISV of SE US rainfall.

**Fig. 6:**
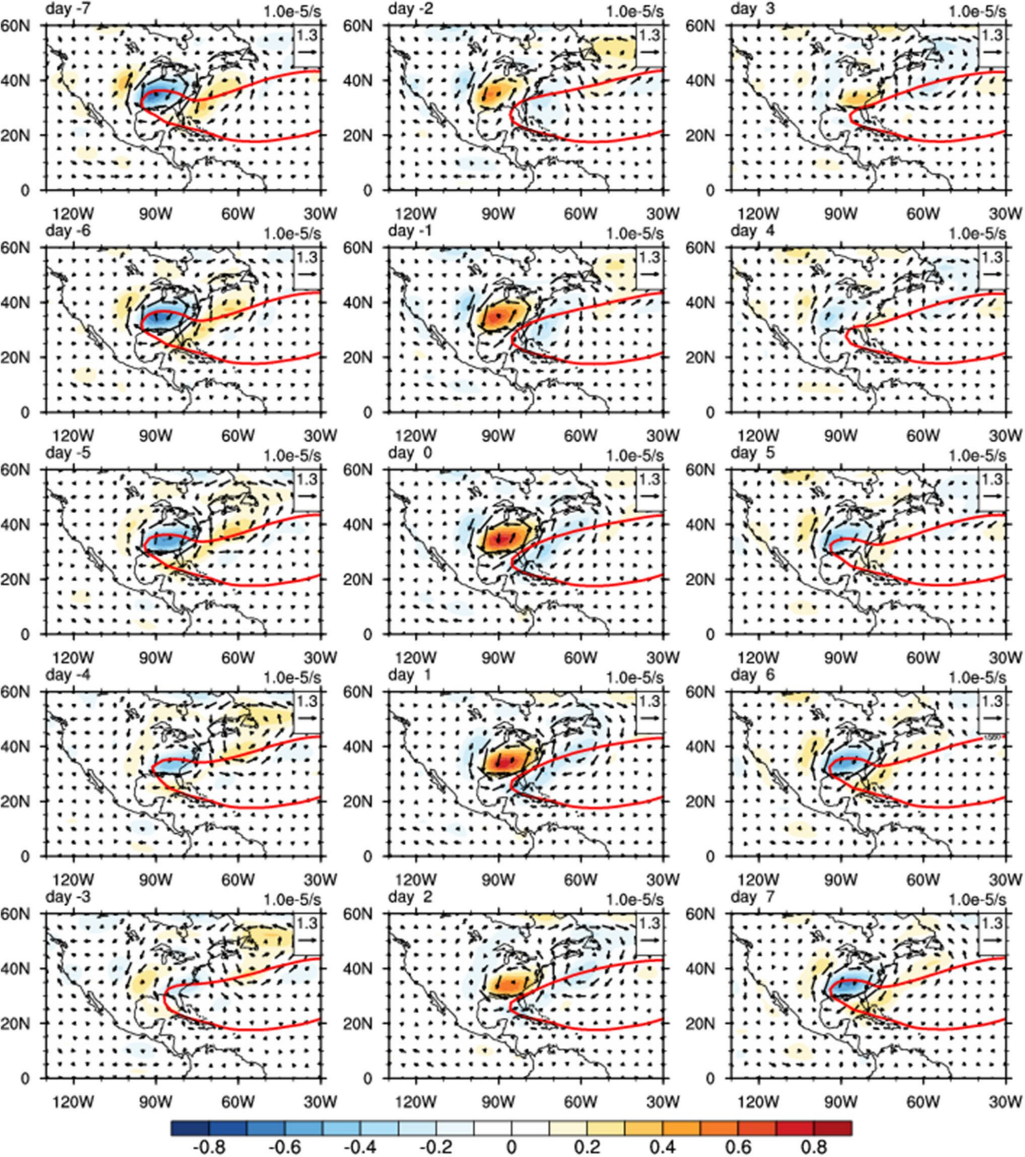
Same as Fig. 5 but for vorticity anomalies (shaded; 10^−5^ s^−1^) and horizontal winds anomalies (vectors; m s^−1^) at 850 hPa. The 1560-gpm isopleth is also plotted (red contour) as the NASH boundary following Li et al. (2011)

Figure 6 shows the composite anomalies of 10‒20-day filtered horizontal wind and vorticity at 850 hPa associated with the ISV of SE US rainfall. During the wet phase from day − 3 to day 3, an anomalous cyclone associated with positive vorticity anomalies is located over the Southeast, which is favorable for excessive rainfall in the region. On the contrary, during the dry phases from day − 7 to day − 4 and from day 4 to day 7, an anomalous anticyclone and negative vorticity anomalies are dominant in the SE US, suppressing the summer rainfall. The intraseasonal evolution in circulation anomalies is consistent with that of the SE US rainfall. During the dry (wet) phase, the anomalous anticyclone (cyclone) over the SE US indicates a westward extension (an eastward retreat) of the NASH (Fig. 6). The southerly (northerly) anomalies in the western flank of the anomalous anticyclone (cyclone) imply a strengthened (weakened) LLJ. Figure 6 suggests that the ISV of SE US rainfall is closely related with the ISVs of NASH and the central US LLJ.

The relationship among the NASH, the central US LLJ, and SE US rainfall can also be seen in Fig. 4, which shows the temporal evolutions of standardized rainfall index (blue line), NASH intensity (red line) and its western ridge location (grey bars), and the jet strength over the central US (green line). Clearly, the NASH intensity leads the LLJ by 3 days; the latter leads the SE US rainfall by 1 day, respectively. Result from a lead-lag correlation analysis also illustrates that the central US LLJ leads the SE US rainfall by 1 day with a maximum correlation coefficient of − 0.40, exceeding the 0.01 significance level. Given the concomitant variations of the NASH western ridge and the LLJ intensity (Fig. 4), the high-pressure western ridge also leads the SE US rainfall by 1 day. These results suggest that the ISV of NASH intensity may modulates the central US LLJ, which may further have impact on the ISV of SE US rainfall.

Figure 4 also indicates that the highest precipitation over the SE US (day 0) leads the NASH by about 2 days (before the NASH reaches its maximum intensity on day 2). Three days later, the LLJ index is at the highest (day 5). This feature implies that the ISV of southeast summer rainfall may also provide feedbacks to affect the NASH, and the central US LLJ subsequently. The east–west shift of the western ridge of NASH is almost consistent with the evolution of the jet, indicating a close interaction between the NASH and the central US LLJ. In the next section, we will study the mechanism for the three-way interaction among the NASH, the central US LLJ, and the SE US rainfall in details.

### Mechanism for the three-way interaction among the NASH, the central US LLJ, and the SE US rainfall on intraseasonal time scales

#### Effect of the NASH intensity on the western ridge movement and the central US LLJ

Spectrum analysis suggests that the 10‒20-day variation is significant in the NASH intensity (not shown). The ISV of NASH intensity is highly correlated with its western ridge and the LLJ strength but with three-day lead (Fig. 4). NASH will change its shape while its intensity varies (Li et al. 2012). When the NASH is weakened from day − 7 to day − 4, its area (defined as the area within 1560-gpm isopleth) becomes to shrink and its western ridge retreats eastward from 95°W to 90°W accordingly (Fig. 6). The zonal pressure gradient is thus reduced over the central US, resulting in a weakening LLJ (Fig. 4). On the contrary, an enhanced NASH not only leads to a westward extension of its western ridge, but also increases the zonal gradient of pressure, which further strengthens the southerly winds over the central US (Fig. 6), although the strengthening of the meridional winds lags the NASH intensity by 3 days.

**Fig. 7:**
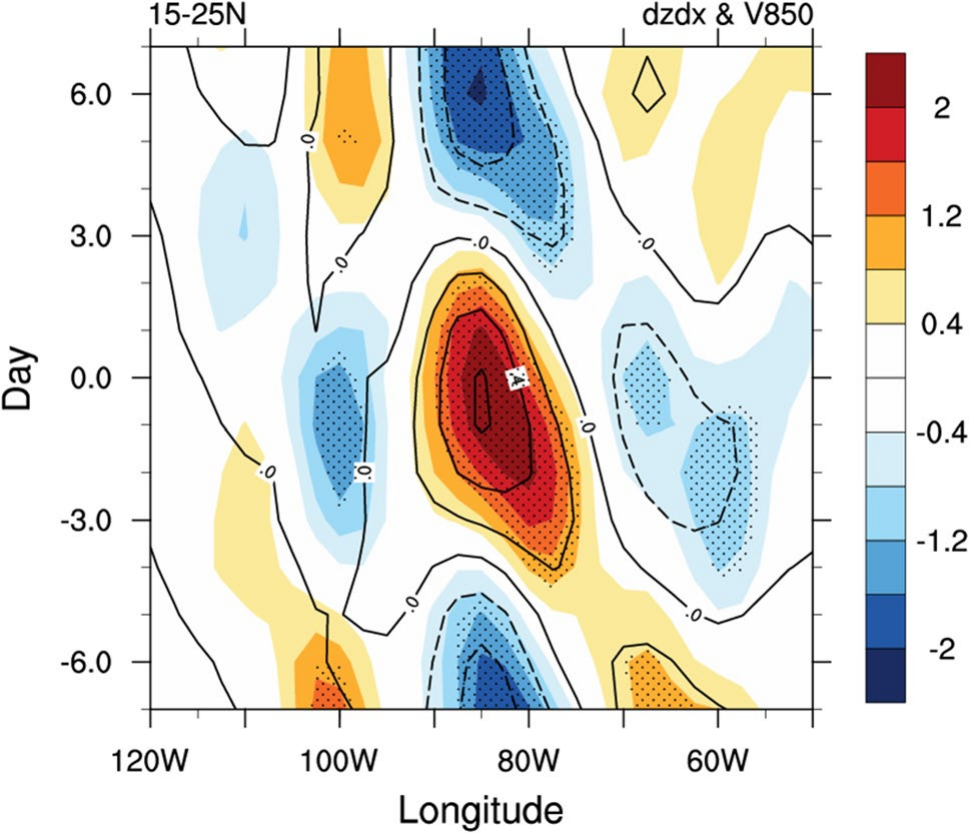
Hovmöller diagram of composite 10‒20-day filtered zonal gradient of geopotential height (shaded; 10^−6^ gpm m^−1^) and meridional wind anomalies at 850 hPa (contours; m s^−1^) along 15º‒25ºN from day − 7 to day 7 based on the SE US rainfall index. Zonal gradient of geopotential height anomalies exceeding 0.05 significance level are highlighted by dots

**Fig. 8:**
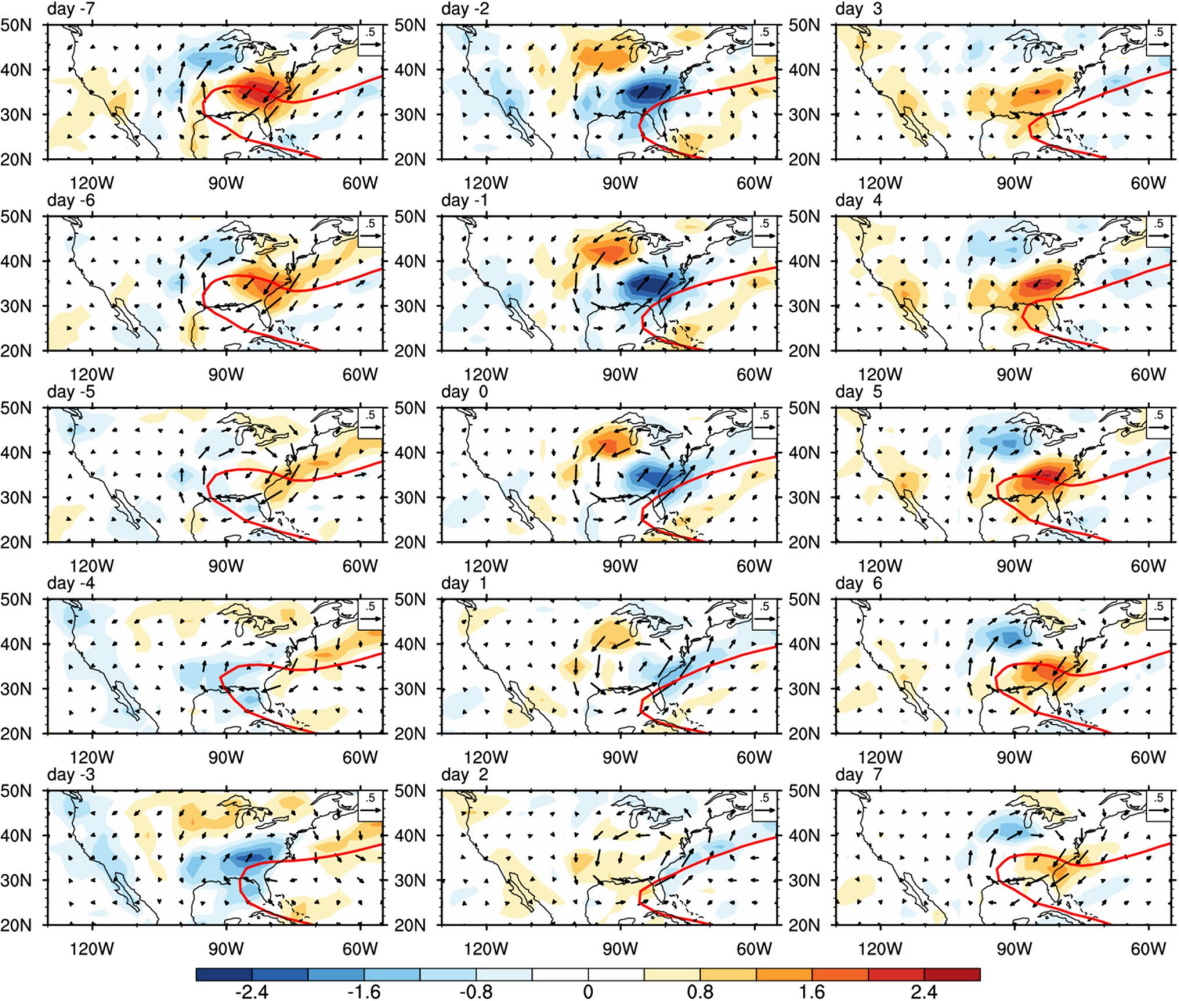
Same as Fig. 6 but for column-integrated moisture flux divergence anomalies (shaded; 10^−7^ kg m^−2^ s^−1^) and column-integrated moisture flux anomalies (vectors; kg m^−1^ s^−1^)

Instead of the synchronous variations of the intensity of the NASH and its western ridge on the interannual, interdecadal or even longer time scales, our result suggests a lag relationship between the ridge location and the NASH intensity on the quasi-biweekly time scales. Such a lag relationship can also be observed in Fig. 7, which shows the westward propagation of the zonal gradient of geopotential height associated with the ISV of the NASH intensity along the southern flank of the subtropical high-pressure system. When the NASH intensity increases from day − 4 to day 2 (Figs. 4, 8), positive zonal gradient of the 850-hPa geopotential height and associated southerly wind anomalies in the southern flank of NASH propagate westward from 70°W to 90°W in the region to the west of the NASH center (Fig. 7). Such southerly wind anomalies continue to move westward to 100ºW from day 2 to day 5, then are forced to turn northward on encountering the eastern slope of the North American Cordillera. This northward flowing air column has to gain anticyclonic vorticity (ξ) in order to compensate for the increase of planetary vorticity (*f*) and an anticyclonic shear in the northward component of the wind must develop due to the potential vorticity (PV) conservation (Ting and Wang 2006, Pu et al. 2016). We thus observe intensification of the LLJ (Figs. 4, 8). On day 5, the LLJ reaches its maximum intensity, 3 days after the NASH is strongest (Fig. 4). Similarly, from day 2 to day 7, when the NASH intensity decreases, northerly wind anomalies associated with the negative zonal gradient of geopotential height propagate from 70°W to 90°W along the easterly trade winds in the southern flank of NASH, and weaken the LLJ starting from day 6. Ting and Wang (2006) pointed out that the trade winds along the southern flank of the NASH played a dominant role in the maintenance and the interannual variation of the LLJ over central US. Our results further suggest that the ISV in NASH intensity propagates westward on the equator side of the NASH and exerts an impact on the LLJ.

#### Effects of the central US LLJ and the NASH western ridge on the SE US rainfall

Figures 4 and 6 demonstrate that 1 day before the maximum rainfall over the SE US, the LLJ index reaches its minimum and the western ridge of NASH retreats to its easternmost position at about 85ºW. How the LLJ and the NASH western ridge modulate the Southeast rainfall will be studied in this subsection.

Figure 8 shows the evolutions of moisture flux and moisture flux convergence from day − 7 to day 7. On day − 7, the moisture flux converges in the central US and diverges over the Southeast. In the meantime, the western ridge of the NASH reaches its westernmost position at 95ºW and the entire SE US is controlled by the high-pressure system. The southerly wind anomalies along the western flank of NASH tend to strengthen the LLJ and enhance the moisture transport from the Gulf to higher latitudes, leading to abnormally high rainfall over the northern central US. The SE US, on the contrary, is in the dry phase because of moisture divergence in the region (Fig. 8). From day − 7 to day − 5, both of the moisture convergence over the central US and the moisture divergence over the SE US are reduced associated with the weakened jet and the eastward shifted NASH (Fig. 8). On day − 4, the center of moisture convergence moves southward accompanying with the further weakening of the LLJ, and the jet index becomes negative (Fig. 4). At the same time, the western ridge of NASH shifts eastward quickly to reach at 91ºW (Fig. 8). On day − 3, the southerly winds and associate moisture transport along the west flank of NASH move eastward accompanying with further eastward retreatment of the NASH, and moisture converges into the SE US. On day − 2, an anomalous cyclone is clearly formed over the Southeast (Fig. 6). The enhancement of this anomalous cyclone causes more atmospheric moisture to converge into the SE US (Fig. 8) and keeps the western ridge of NASH in a southeast position from day − 2 to day 0 (Fig. 4). The jet index reaches its minimum on day − 1 (Fig. 4). The anomalous cyclone is weakened and moves eastward from day 0 to day 3 (Fig. 6). From day 3 to day 4, the jet index becomes positive (Fig. 4), the anomalous cyclone is replaced by an anomalous anticyclone over the SE US, and the moisture flux converges to the northern central US again (Fig. 6). On day 5, the anticyclone intensifies associated with the enhanced LLJ and leads to a suddenly westward shift of the NASH western ridge to 95°W. The NASH controls the SE US again and suppresses the rainfall over the region.

The ISV of LLJ could impact rainfall over the central US directly and over the SE US indirectly. The strengthened (weakened) LLJ transports more (less) water vapors into the northern central US and results in abnormally higher (lower) rainfall over the region (Fig. 5). The increased (decreased) latent heating associated with the rainfall can enhance an anomalous cyclone (anticyclone) in the northern central US and further trigger an anomalous anticyclone (cyclone) to its east at the lower level (Hoskins 1991; Wu and Liu 2003; Wei et al. 2014, 2015). The anomalous cyclone (anticyclone) in the northern central US weakens (enhances) the LLJ to its west, and the anomalous anticyclone (cyclone) over the SE US favors a southeastward shift of the dry (wet) condition.

In summary, the ISV of the LLJ modulates the moisture flux over the central US. An anomalously strong LLJ usually facilitates moisture convergence and positive precipitation anomalies over the central US, leading to the enhanced condensational latent heating and an anomalous cyclone and anticyclone in the central US and SE US, respectively. This circulation anomalies influence the shifts of NASH ridge and the moisture transport into the SE US, resulting in rainfall variability over the Southeast.

#### Impact of the SE US rainfall on the NASH intensity

The temporal evolutions of NASH intensity and SE US rainfall show that the NASH maximum intensity lags the highest rainfall over the Southeast by 2 days (Fig. 4), implying a possible effect of the ISV of SE US rainfall on the NASH intensity.

Climatologically, condensational heating associated with summer rainfall plays an important role in the formation and intensity variation of subtropical high-pressure systems (Rodwell and Hoskins 2001; Wu and Liu 2003; Liu et al. 2004, 2007; Miyasaka and Nakamura 2005; Li et al. 2014; Wei et al. 2014, 2015, 2017; Yang and Li 2017a). On intraseasonal time scales, the variation of SE US rainfall leads to that of condensational heating over the SE US, triggering anomalous meridional flows in the lower troposphere along the NASH ridge line (Fig. 6) according to the Sverdrup vorticity balance (Hoskins 1991; Wu and Liu 2003) and thus modulating the NASH strength.

**Fig. 9:**
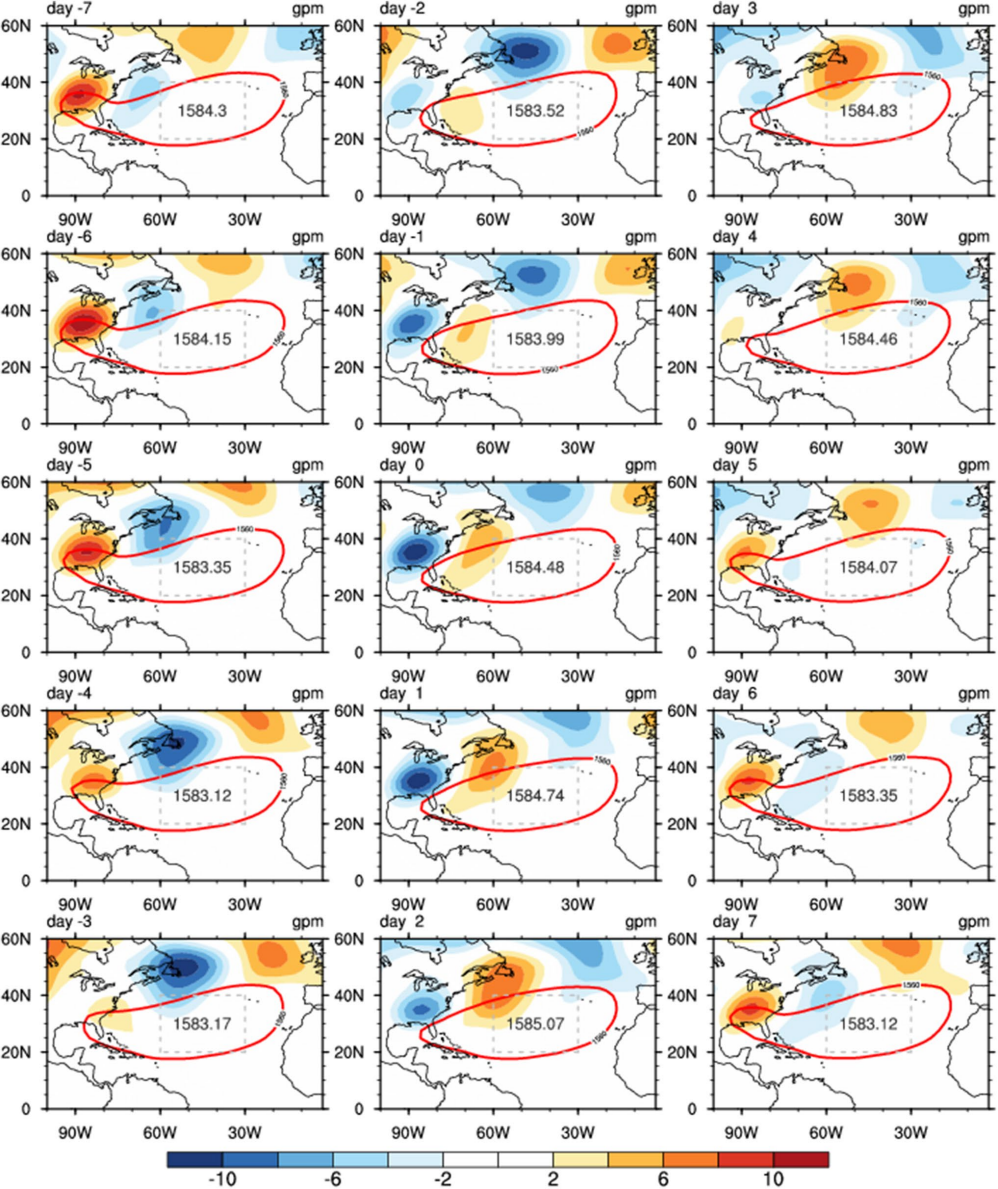
Same as Fig. 6 but for geopotential height anomalies (shaded; gpm) and the NASH (contours; 1560 gpm). The grey numbers are the domain-averaged geopotential heights over the NASH central region (grey dashed boxes, 60°W–30°W 20°N–40°N)

Besides, the rainfall induced condensational heating could also excite wave trains propagating along the great circle path. The intraseasonal perturbations in middle latitudes wave trains affected the ISV of subtropical highs (Lau and Holopainen 1984; Yang and Li 2016b, 2017b). Figure 9 analyzes the ISV of geopotential height anomalies at 850 hPa associated with the evolution of SE US rainfall. A wavetrain-like pattern is observed at the north of the NASH. This wave train originates from the SE US and propagates eastward along the westerly winds to the north of the NASH (Fig. 9). Specifically, on day − 6, the SE US rainfall is minimum (Figs. 4, 5). Negative heating anomalies associated with the rainfall anomalies over the SE US excite an anomalous cyclone to the east and result in negative geopotential height anomalies with its center at about 38°N, 62°W (Fig. 9). This low-pressure center strengthens and moves northeastward from day − 6 to day − 4. When the low-pressure center is located at about 48°N, 54°W on day − 4, the NASH intensity is at its minimum (Figs. 4, 9). On day − 3, this low-pressure center keeps moving northeastward to higher latitudes (about 50°N), and its impact on the NASH becomes weaker.

The NASH begins to gain strength associated with the rainfall increase over the SE US. On day 2, the high-pressure anomaly excited by the anomalous condensational heating associated with positive rainfall anomalies over the SE US is located to the north of NASH with its center at about 45°N, 57°W, which strengthens the NASH to its maximum intensity (Fig. 9). It is also noticed that the positive (negative) height anomalies decrease (increase) over the SE US and the negative height anomalies increase (decrease) to the east with the center at about 60ºW from day − 6 to day − 3 (day 0 to day 3).

Figure 9 also shows a wave train originated from the SE US, associated with the Southeast rainfall and travels along the great circle route (Hoskins and Ambrizzi 1993) over the northern North Atlantic (Fig. 9). It exerts an effect on the NASH intensity and leads to a 2-day lag of the NASH intensity after the ISV of SE US rainfall.

## Conclusion

A significant 10‒20-day ISV of summer rainfall is found over the SE US. Analysis of the temporal evolutions of the SE US rainfall, the central US LLJ, and the NASH indicates that the highest rainfall over the SE US lags the strongest LLJ by 1 day and leads the NASH maximum intensity by 2 days. Then the NASH intensity leads its western ridge and the LLJ by 3 days. The evolutions of the LLJ index and the longitude of the NASH western ridge are almost synchronous.

**Fig. 10:**
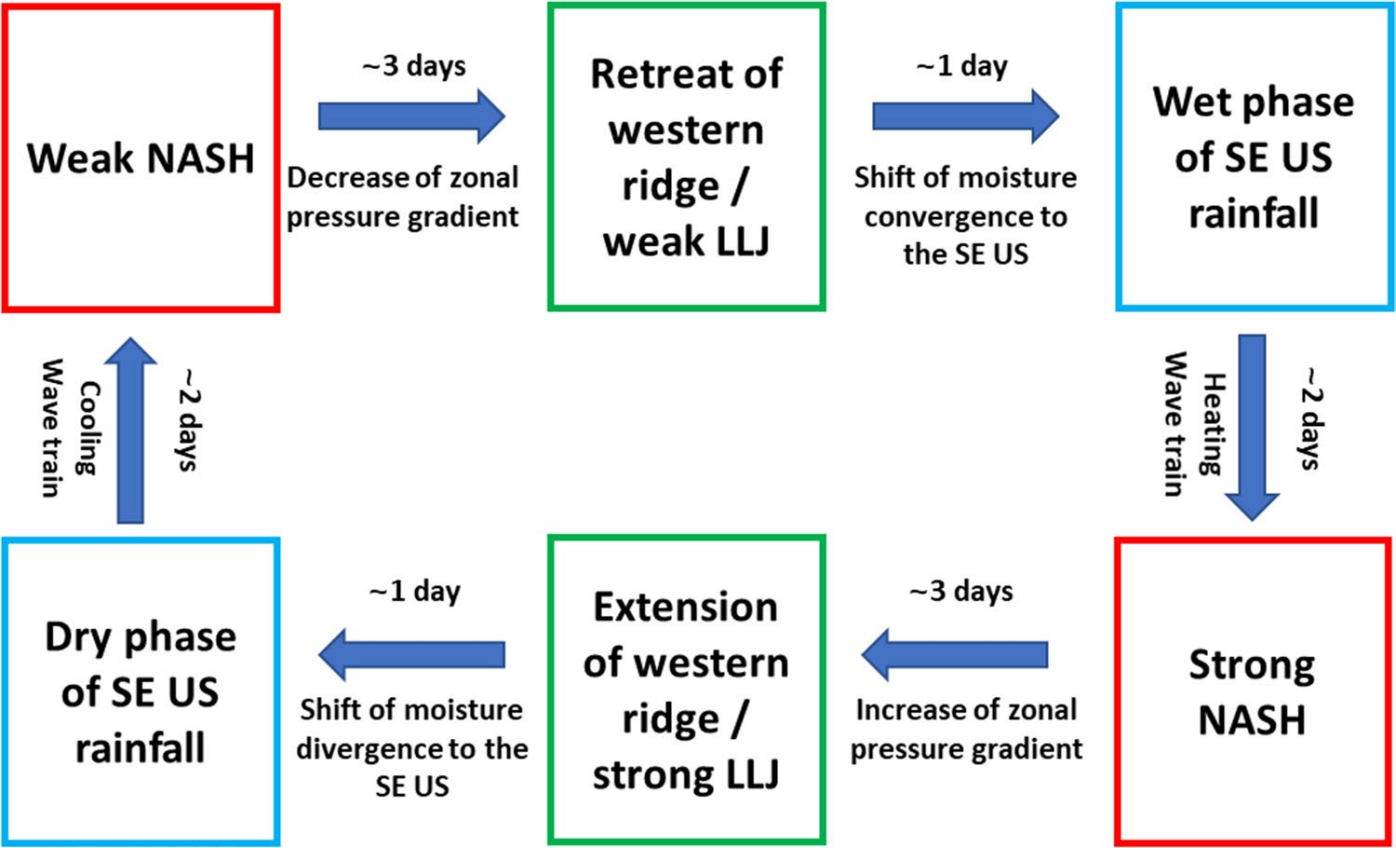
Schematic diagram showing the three-way interactions among the SE US rainfall, the central US LLJ, and the NASH on intraseasonal timescales

The dynamical mechanism underlying these three-way interactive processes is revealed (Fig. 10): 4 days before (day − 4) the strongest rainfall occurring over the SE US (day 0), the NASH is the weakest and its size reaches a minimum. Accordingly, the western ridge of NASH retreats eastward to the east of 90ºW on day − 4. The LLJ is weakened (i.e., LLJ index is negative) due to a decreased zonal gradient of pressure associated with the NASH weakening. As a result, moisture convergence is reduced to the central US. The combined effect of weakened LLJ and the eastward retreat of the NASH western ridge is favorable for the formation of an anomalous cyclone and enhanced rainfall over the Southeast. One day after the LLJ reaches its weakest intensity (day − 1) over the central US, the SE US rainfall reaches its maximum on day 0.

The increased latent heat associated with the abnormally high rainfall over the SE US excites an anomalous anticyclone/negative vorticity to its northeast, and the anticyclone intensity increases while it propagates eastward along the westerly flow to the north of the NASH from day 0 to day 2 (Fig. 6). The positive geopotential height anomaly associated with this anomalous anticyclone enhances the NASH intensity, and contributes the NASH to reach its maximum strength on day 2. The stronger NASH tends to expand, resulting in a westward extension of the NASH western ridge and an enhanced LLJ over the central US. Then an anomalous anticyclone formed over the SE US suppresses the rainfall over the region. After these processes, the SE US turns from a wet phase into a dry phase.

Mo’s study suggested that eastward propagating ISV may impact the SE US rainfall (Mo 2000). Our study demonstrates that the ISV of the SE US rainfall could also be regulated by the ISV of the NASH (i.e., from the east). The latter (ISV signal from the east) propagates westward along the trade wind in the southern flank of the NASH and modifies the LLJ inducing rainfall variations on the intraseasonal scale over the central US, as well as the rainfall over the SE US by the triggered anticyclone/cyclone over the Southeast (ISV signal from the west).

The eastward propagation of the ISV signal over the SE US can further propagate to the North Atlantic following the great circle route and modulate the NASH intensity. Previous studies have investigated the teleconnections of ISV in the Northern Hemisphere (Kikuchi and Wang 2009; Moon et al. 2013) and on more global scales (Kikuchi and Wang 2009). Our results suggest that the ISV of SE US rainfall is an important part of the global ISV although we have focused only on the interactive process among the SE US rainfall, the central US LLJ, and the NASH.

The plausible causal relationship proposed in this paper is consistent with our basic understanding of the summer circulation in this region. A more rigorous proof of the causality demands carefully designed numerical experiments and further statistical analysis, which will be our upcoming tasks. The result of this study suggests that improved prediction of the SE US summer rainfall across intraseasonal scales depends critically on the model representation of three-way coupling among the NASH, the central US LLJ, and the SE US rainfall. The high predictability of NASH may open a pathway to ameliorate the SE US rainfall prediction in summer.
